# Amplatz versus Balloon for Tract Dilation in Ultrasonographically Guided Percutaneous Nephrolithotomy: A Randomized Clinical Trial

**DOI:** 10.1155/2019/3428123

**Published:** 2019-01-03

**Authors:** Hamid Pakmanesh, Azar Daneshpajooh, Mahboubeh Mirzaei, Armita Shahesmaeili, Morteza Hashemian, Mohsen Alinejad, Ali Asghar Ketabchi, Ali Tavoosian, Mohammad Reza Ebadzadeh

**Affiliations:** ^1^Department of Urology, Shahid Bahonar Hospital, Kerman University of Medical Sciences (KMU), Kerman, Iran; ^2^Department of Epidemiology, Kerman University of Medical Sciences (KMU), Kerman, Iran; ^3^Department of Anesthesiology and Pain Medicine, Shahid Bahonar Hospital, Kerman University of Medical Sciences (KMU), Kerman, Iran

## Abstract

**Purpose:**

To compare balloon with Amplatz for tract dilation in totally ultrasonographically guided PCNL (UPCN).

**Methods:**

We randomized 66 patients candidate for sonographically guided PCNL in the flank position in two study groups. In the first group, we used single step Amplatz dilation (AG) technique in which the 28- or 30-French Amplatz dilator is used for tract dilation. In the other group, we dilated the tract using balloon dilator (BG). We compared procedure time, success rate of dilation, and postoperative clinical outcomes and cost between two groups.

**Results:**

The rate of short dilation was higher in the Amplatz group (57.6%) compared with Balloon group (36.4%) (P=0.08). When using Amplatz for lower pole access, short dilation occurred in 81% of cases compared with 44% in the BG (P=0.02). Overall operation was longer in the AG (80±21 versus 65±20 minutes P=0.02). Stone free rate was 87.9% in the AG compared with 72.7% in the BG (p=0.12). Mean cost of the surgery was 603±85 USD and 718±78 USD in the AG and BG, respectively (P=0.0001). Hemoglobin drop, transfusion rate, renal function alteration, duration of hospitalization, and complication rate based on Clavien classification were similar in both groups.

**Conclusions:**

AG showed a higher rate of short dilation compared with BG; consequently, overall operating time was significantly longer in the AG whereas BG was significantly more expensive than AG. Bleeding and other complications were similar in two groups. We observed an advantage for balloon dilation over Amplatz when approaching the lower pole calyxes.

## 1. Introduction

Nowadays, percutaneous nephrolithotripsy (PCNL) is the first-line approach for the treatment of kidney stones larger than 20 mm [1]. Fluoroscopy is the most popular method to guide percutaneous access. Some urologist introduced ultrasonography to reduce radiation exposure during needle puncture. Recently, completely ultrasonographically guided PCNL (UPCN) has been presented, with acceptable success rate and complication, to eliminate radiation hazard [2–7].

Normal kidney has some mobility in the retroperitoneum; this fact is a challenge in dilation of the access tract during UPCN using Amplatz dilator especially in the single step technique. During UPCN, the instruments are less visible than fluoroscopy guided PCNL; thus the Amplatz dilator may push the kidney and do not enter the calyx. This state is called “short” dilation. Previous reports have shown a relatively high rate of short dilation during UPCN using single step Amplatz dilation [3]. Theoretically, balloon dilator has lower rate of short dilation; however, its use is limited due to higher cost. We designed this trial to find out whether balloon dilator has higher success rate compared with Amplatz dilator for tract dilation in UPCN. To the best of our knowledge, this is the first randomized clinical trial concerning this issue.

## 2. Method

This clinical trial had been registered at 2016-05-25 before being started. The registration code is IRCT2016041727426N2.

**Figure 1:**
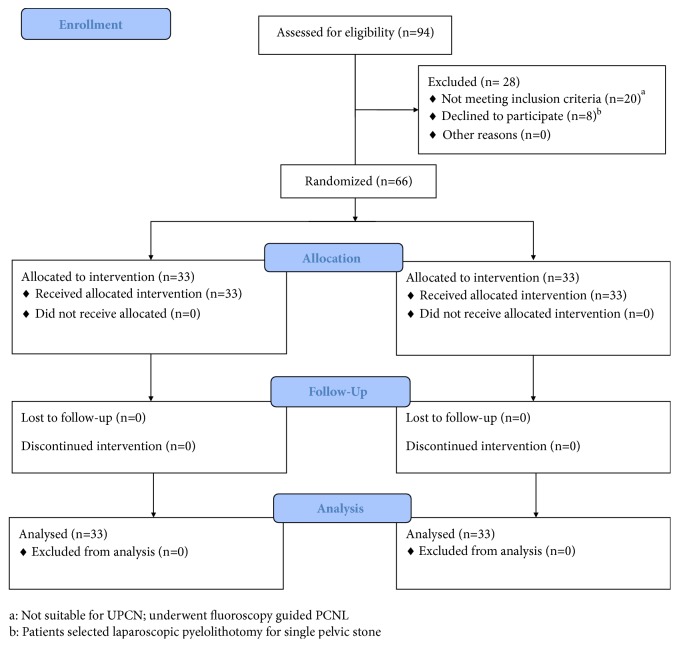
CONSORT flow diagram of the study.

*Inclusion Criteria and Participants.* From July 2016 to December 2016, 94 consecutive patients who were referred to our clinic with kidney stones larger than 20 mm or smaller stones resistant to SWL were assessed for eligibility. We included all cases with single pelvic or calyceal stones, partial or complete staghorn. Patients who had small multiple opaque stones in different calyxes who were suitable for fluoroscopically guided PCNL (20 patients) were excluded. Eight patients with single pelvic stone chose laparoscopic pyelolithotomy. There was no patient with uncorrected coagulopathy. Finally, 66 patients were selected and allocated in two study groups with simple randomization method (Figure 1).

### 2.1. Surgical Procedure

A single surgeon experienced in UPCN with more than 250 PCNL procedures per year (first author) performed all operations.

*Patient Preparation and Positioning.* Urine culture was performed for all patients and infection was treated. All patients received prophylactic antibiotic. General anesthesia was inducted for all patients. First, a five French ureteral catheter was inserted using cystoscope in the lithotomy position. Then, we repositioned the patient to the flank position and placed a cushion under the contralateral flank. More, the table was flexed at the umbilicus level to produce a wide area for access between lower rib and iliac crest to facilitate subcostal access in all cases.

**Figure 2:**
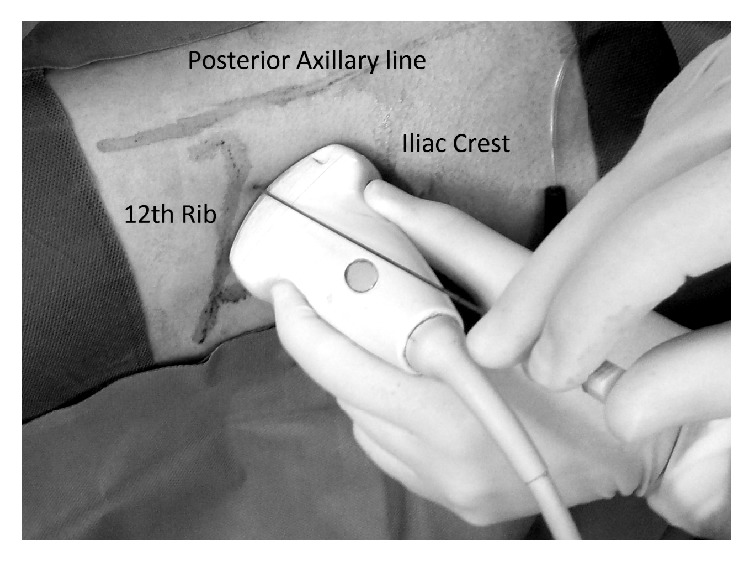
Needle insertion for right kidney puncture in flank position.** The probe is placed longitudinally parallel to the longitudinal axis of the kidney, which runs obliquely downward and laterally. The needle is inserted adjacent to the probe using a free-hand technique without using needle holder guide.**

*Ultrasonography Guided Puncture.* The area of puncture was between the quadratus lumborum muscle as the medial border, posterior axillary line as the lateral border, the 12^th^ rib as the superior border, and the iliac crest as the inferior border (Figure 2). All accesses were subcostal. We used a two-dimensional convex shaped 3.5 MHz abdominal ultrasound probe and placed the probe longitudinally parallel to the longitudinal axis of the kidney, which runs obliquely downward and laterally. To obtain as shorter as possible access tract to the kidney, Chiba needle was inserted parallel to the probe using free-hand technique without the needle holder guide (Figure 2). We observed the needle tip entering the desired calyx. To confirm accurate placement of the needle, the needle was aspirated using a 10 cc syringe while injecting saline to the ureteral catheter. Then, a 0.035-inch J-tip 150 cm guide wire was placed in the system through the needle.

*Tract Dilation.* The length of the tract from the skin to the desired calyx was marked on the Chiba needle and recorded for use as a guide for all future steps of tract dilation. Then, we dilated the tract up to 11-Fr using fascial dilators. At this stage, in the first study group, we used a 28 or 30 Fr Amplatz Type dilator (Boston Scientific US) for single step tract dilation without sequential tract dilation (known as one-shot technique in fluoroscopically guided PCNL [8]). In the other study group, we used a 28 or 30-Fr high-pressure balloon dilatator (Boston Scientific US). Then, the working sheath was inserted over the Amplatz or balloon to the desired calyx. Finally, the Amplatz dilator or balloon was removed and the working sheath and the guide wire left in place.

*Nephroscopy and Stone Extraction.* A 20.8 Fr, 12° Richard Wolf nephroscope, and pneumatic Swiss LithoClast® EMS set was used for stone disintegration and extraction. At the beginning of the nephroscopy, if we noticed that the working sheath had not reached the desired calyx (short dilation), we followed the guide wire and entered the system using a biprong forceps for dissection under direct endoscopic inspection [9, 10]. The entire calyceal system was explored for the presence of the stones using rigid nephroscope and finally we looked for any possible residual stone using ultrasonography. At the end of the operation, the surgeon chose appropriate exit strategy for each patient individually. A Double-J stent was inserted antegradely for patient with single functional kidney, those with history of urinary tract infection or marginal renal function, or in case the pyelocalyceal system was injured significantly during the procedure or there was significant bleeding. We left a 20 Fr Foley catheter in place if significant bleeding was present. In the uneventful operations, tubeless PCNL was preferred.

### 2.2. Clinical Assessments

The patient and the physician who recorded the pre- and postoperative data as well as the statistical analyzer were all blind regarding the study group.

*Preoperative Data.* At the preoperation night, we checked the blood hemoglobin concentration (Hb as g per dl) and serum creatinine level (Cr as mg per dl). We measured stone dimensions using the preoperative noncontrast enhanced computed tomography scan of the patient using digital calipers (Table 1).

*Intraoperative Data.* One of the operating room staffs recorded the time spent for each step of the surgery separately using a stopwatch.

The place of the working sheath relative to the desired calyx at the beginning of the nephroscopy was reported as appropriate, short, or too far. If the guide wire became kinked or pushed out of the collecting system and insertion of the Amplatz sheath in the collecting system was failed, dilation recorded as failed. In this case, more attempts for needle puncture and tract dilation were made. The frequency of the attempts was recorded.

*Postoperative Data.* A blood sample was obtained at 5 a.m. on the first postoperative day and was sent to the laboratory for testing the Hb and Cr level. Hb drop was defined as the difference between preoperative and 24-hour postoperative hemoglobin concentrations. The cut-off level for blood transfusion was an Hb concentration less than 10 g/dl. The timing of the transfusion and total transfused units as well as the minimum Hb level for each patient was recorded. We calculated alteration in renal function by subtracting postoperative estimated GFR (after 24h) from preoperative GFR. Stone free status was evaluated using kidney ultrasonography as well as X-ray KUB at the first postoperative day. We prospectively recorded any complication using a checklist based on standardized Clavien-Dindo grading system for complications in PCNL [11].

### 2.3. Statistical Analysis

The Chi square tests and Fisher exact test were used to compare the frequencies in two arms. To compare the means, student T-tests and Mann–Whitney U test were used. Data were presented as relative frequencies and percentage for categorical as well as mean (standard deviation (SD)) for continuous variables. All P-values were two-sided and values less than 5% were considered as statistically significant. SPSS 22 software was used (IBM SPSS statistic USA) to analyze the data.

## 3. Results

### 3.1. Demographic and Baseline Characteristics

There were no statistically significant differences between two groups regarding demographic and baseline characteristics (Table 2). Most patients (33.3 %) had staghorn stone followed by single pelvic stone (28.8 %) and single calyceal stone (24.2%), and 13.6 percent had more than one calyx involved by stones. The mean calculated stone surface area was 587 ± 457 mm^2^ (range from 141 to 1883 mm^2^). The stone burden and distribution was not different between two groups (Table 2).

### 3.2. Clinical Outcomes

Most important clinical outcomes are summarized in Table 3.

#### 3.2.1. Access Status

The rate of short dilation was higher in the Amplatz group (57.6%) compared with Balloon group (36.4%) (P=0.08) (Table 3). The overall rate of short dilation was 61% when approaching the lower pole calyxes compared with middle (18%) or upper pole calyxes (40%) (P=0.01). When using Amplatz for lower pole access, short dilation occurred in 81% of cases compared with 44% in the BG (P=0.02).

Overall 18/66 (27%) of patients had a history of previous operation including open stone surgery or PCNL (Table 2). Short dilation occurred in 5/10 (50 %) of these cases in the AG compared with 2/8 (25%) in the BG (P=0.12). For patients who had not a history of surgery, this rate was 14/23 (60%) in the AG and 10/25 (40%) in the BG. In two patients in the AG and three patients in the balloon group, too far dilation from the desired calyx occurred.

Most operations were accomplished with a single access (93.9% in AG and 90.9 % in BG (P=1)) (Table 3). In one patient in the AG, the access was established in the renal pelvis. In this patient, we removed the stone completely and placed a Double-J stent. In follow up, no complication occurred.

There was no significant difference comparing tract dilation time between Amplatz with BG (Table 3). Nephroscopy time was longer in the Amplatz group (AG) than the balloon group (BG). This difference did not reach the statistical significance (P=0.10); however, when the overall operating time was considered, the AG had significantly longer time than BG (80.61±21.28 versus 65.91±20.60, minutes, P=0.02).

#### 3.2.2. Stone Free Rate

Early stone free rate which was evaluated on the first postoperative day was 87.9% in the AG and 72.7% in the BG (p = 0.12). Only one patient in the BG who had a nonopaque single pelvic stone of 628 mm^2^ showed a 23-mm residual stone in the middle calyx postoperatively that was suitable for second look PCNL. Other residual stones were appropriate for SWL (4/33 (12%) in the Amplatz and 8/33 (24%) in the BG)

#### 3.2.3. Complications

Table 4 summarizes all complications based on standardized Clavien-Dindo classification for complications in PCNL. Overall, both group showed similar rate of postoperative complication (33.3%) (P=1).

*Fever.* Fever more than 38.5°C was detected in seven patients (21.2%) in the AG and in six patients in the BG (18.2%) (P=0.75). Only in one patient of the AG, fever did not resolved with conservative treatment until we placed a Double-J stent at the fourth postoperative day (Clavien grade IIIa).

*Transfusion Rate.* Median Hb drop was 1.2 g/dl in the AG and 1.9 g/dl in the BG (p=0.8). The time of transfusion was 1.4±0.5 day postoperatively for the AG and 0.5±0.5 for the BG (p=0.04) (two patients received blood intraoperatively). We did not encountered perioperative bleeding requiring quitting the operation.

*Leak.* Two patients in each group complained of urinary leakage less than 12 hours from the nephrostomy tract after nephrostomy removal; however, none suffered constant leak for more than 48 hours.

*Other Complications.* In a 27-year-old man with staghorn in the BG, a chest tube was placed under local anesthesia due to symptomatic hydrothorax (Clavien score IIIa). Other complications did not happened in this cohort.

### 3.3. Cost

Total cost of the procedure was calculated for each patient (Table 1). For the AG mean cost was 603±85 USD (490 to 840 USD) that was significantly cheaper than the BG (718±78 USD, 630 to 890 USD) (P=0.0001).

## 4. Discussion

This study is the first randomized clinical trial comparing two dilation techniques in UPCN. A single experienced surgeon performed all operations; data and complication rate as well as success rate were recorded blindly. In the present study on patients undergoing UPCN in the flank position, we found that balloon dilation shows a relatively lower rate of short dilation compared with single step Amplatz dilation technique. Consequently, overall operating time was significantly shorter in the BG. However, the operative cost was significantly higher in the BG.

Short dilation is secondary to the mobility of the kidney. Consequently, during dilation, the tip of the Amplatz dilator or the working sheath may fall out of the system or the Amplatz sheath does not enter the kidney and even the guide wire becomes kinked or drops out of the system. Formerly we were using the aiming device for needle puncture but the access length was longer with this device compared with free-hand technique. Our experience shows that shorter access length is associated with higher dilatation success. As depicted in the Figure 2, in our method, with the needle parallel to the probe at the middle of it, the access length will be as short as possible. In addition, it is easier to access upper pole calyxes using this method. During tract dilation, we use ultrasound as guide to follow the balloon or Amplatz dilator entering the kidney. The guide wire is echogenic whereas the dilators are not. To make sure that the dilator is entered the calyx, we monitor the echogenicity of the guide wire, which disappears when the dilator is passing over it. In some cases, especially in the obese patients or while obtaining the second access, it is difficult to observe with ultrasound guide the balloon or Amplatz dilator entering the calyx. Therefore, we trust on the measurement of the needle depth as well as the tactile sensation during dilation process that is the feeling of the dilator progression over the guide wire and passing through different tissue layers. Finally, if we encounter a short dilation, we apply a salvage technique that has been reported previously by Lezrek et al. [10]. In this technique, we use a biprong forceps to enter the calyceal system and then introduce the Amplatz sheath to the system with a twisting motion over the nephroscope [9]. This technique may also be helpful even in fluoroscopy guided PCNL in case of short access when contrast material is spread around the kidney and it is difficult to see the collecting system under the fluoroscopy [9]. Further, when there is sever scar tissue around the kidney this technique may be helpful. The alternative approach in cases with short dilation is to insert again the Amplatz dilator or balloon over the guide wire and try to dilate the tract and insert the working sheath over it. However, in our experience this decision is sometimes associated with access failure or too far dilation. Therefore, we prefer the aforementioned safe and successful salvage technique of tract dilation under direct vision. Recently Shah AK et al. introduced a new dilator with transparent shaft that allows passing a 12F nephroscope within its lumen so that the surgeon can observe the dilation process with direct vision and make sure that the nephroscope is correctly placed in the collecting system [12]. This technique will help to reduce radiation exposure during fluoroscopy guided PCNL and increase dilation success during sonographically guided PCNL as well.

In our study, the rate of short dilation was related to the calyx chosen for access. The rate of short dilation was significantly higher in lower pole approach compared with the middle or upper pole approach. This finding is secondary to the fact that that the lower pole of the kidney is relatively mobile in the retroperitoneum. We observed an advantage for balloon dilation over Amplatz when approaching the lower pole calyxes; in cases with lower pole approach, short dilation occurred in 44% of cases in the BG compared with 81% in the AG. The lowest rate of short dilation was for cases with access to the middle calyxes (18%). Similarly, Song et al. retrospectively compared results of lower, middle, and upper calyx accesses for UPCN. They showed that middle calyx access is faster with higher success rate in UPCN [10]. This may be because when obtaining access to the middle calyx, the access line is perpendicular to the kidney and the forces during dilation of the tract will not rotate the kidney.

Many studies have compared the tract dilation methods during fluoroscopy guided PCNL [13–16] whereas there are only two retrospective studies concerning tract dilation methods in UPCN in the literature [17, 18]. In a retrospective study, Ren Minghua et al. compared the results of two tract dilation methods in UPCN. With a threshold of 9 g/dl, transfusion rate was 27% in the sequential Amplatz dilation group that was significantly higher than the BG (13%). Likewise, Hb drop was significantly higher in the sequential AG (3.5 versus 1.7). They speculated that this difference is a consequence of friction of serial dilators with the renal parenchyma or is a result of false passages during serial Amplatz dilation [17]. Likewise, Zhou et al. in a retrospective study on cases underwent UPCN found that serial Amplatz dilation is associated with a higher rate of transfusion compared with balloon dilation in the hand of less experienced surgeons [18]. In contrast, we did not find any difference between two groups regarding Hb drop (1.7 versus 1.8) or transfusion rate with threshold of 10 g/dl (15% versus 12%).

Dilation in the dense scar tissue is difficult in patients who had a previous history of open or percutaneous renal surgery; however, in these patients, kidney is somewhat fixed in the retroperitoneum and this help the dilation process during UPCN. In our study, we had 12 cases with previous history of renal surgery. In these patients, we encountered less cases of short dilation compared with nonoperated patients in both groups especially in the BG (25% versus 40%). We had no case of dilation failure in this cohort. This finding is different with the results of Andrew et al. study who reported a failure rate of 25% for X-ray guided balloon dilation in previously operated patients [19].

## 5. Conclusions

AG showed a higher rate of short dilation compared with BG; consequently, overall operating time was significantly longer in the AG whereas BG was significantly more expensive than AG. Bleeding and other complications were similar in two groups. We observed a significant advantage regarding appropriate dilation for balloon dilation over Amplatz when approaching the lower pole calyxes.

## Figures and Tables

**Table 1:** Definition of some important variables.

***variable***	***definition***
**Glomerular Filtration Rate **	For adult patients, Modified Diet for Renal Disease (MDRD) equation: GFR (ml/min/1.73 m^2^) = 175 × (Cr in mg/dl) ^−1.154^ × (Age) ^−0.203^ × (0.742 if female)
	For children under 18 years, Bedside Schwartz equation: GFR (ml/min/1.73 m^2^) = (0.41 × Height in cm) / Cr in mg/dl
**Stone Surface Area**	Length (mm) *∗* wide(mm) *∗* *π* *∗* 0.25 (*π* = 3.14159)
**Needle Puncture Time**	The time to get access to the desired calyx under ultrasonography guide using Chiba needle to inserting the guide wire
**Tract Dilation Time**	The time spent to complete dilation using either Amplatz dilator or balloon including inserting the Working sheath
**Access Time**	Total of Needle puncture time and Tract dilation time
**Total Nephroscopy Time**	All the time from the beginning of the nephroscopy, lithotripsy and stone extraction to the moment that the nephrostomy site is closed or the nephrostomy tube is secured
**Overall Operating Time**	All duration of the operation including the time spent for patient preparation and ureteral catheter placemen and repositioning to the end of operation
**Significant Fever**	An oral temperature higher than 38.5°C
**Urine Leakage**	The presence of urine draining on the flank more than 48 hours after removal of the ureteral catheter or nephrostomy tube
**Stone Free Rate (SFR)**	The absence of any residual stone fragment bigger than four millimeters in the ultrasonography or plain radiographic imaging on the first postoperative day
**Cost**	sum of devices, surgical supplies and bed costs from admission to discharge including readmissions

**Table 2:** Baseline characteristics of study participants.

***Variables (unit)***	***Amplatz (n=33)***	***Balloon (n=33)***	***P-value***
**Gender **			0.8
Male	18(54.5)	17( 51.5)	
Female	15(45.5)	16(48.5)	
**Age (years)**	47.39 ± 15.11	47.21 ± 17.13	0.96
**BMI (kg/m** ^**2**^ **)**	26.15 ± 5.78	27.26 ± 6.05	0.44
**Waist diameter (cm)**	93.79 ± 14.19	96.64 ± 10.89	0.42
**Pre-op GFR (mL/min/1.73 m** ^**2**^ **)**	70.2 ± 25.3	71.5 ± 34.1	0.85
**Previous intervention ** ^a^			
Open Stone Surgery	7(21.2)	7(21.2)	1
SWL	13(39.4)	12(36.4)	0.8
PCNL	5(15.2)	2(6.1)	0.23
**laterality**			
Right	14(42.4)	20(60.6)	0.13
left	19(57.6)	13(39.4)	
**Opacity**			
Opaque	29(87.9)	25(75.8)	0.2
Non-opaque	4(12.1)	8(24.2)	
**Stone burden**			
Stones in two calyxes	6(18.2)	3(9.1)	0.32
single pelvic stone	7(21.2)	12(36.4)	
staghorn	10(30.3)	12(36.4)	
single calyceal stone	10(30.3)	6(18.2)	
**Stone area (mm** ^**2**^ **)**	596 ± 473	578 ± 448	0.87

Data presented as frequency (column %) or mean ± SD.

a: three patients had history of both previous PCNL and OSS.

**Table 3:** Comparison of clinical outcomes in between Amplatz and balloon UPCN groups.

***Variables***	***Amplatz (n=33)***	***Balloon (n=33)***	***P***
**Tract length, cm**	7.92 ± 2.40	8.22 ± 1.95	0.57
**Needle puncture time, minutes **	6.11± 6.82	4.58±3.68	0.98^a^
**Tract dilation time, minutes **	5.22±5.63	5.70±6.24	0.67^a^
**Total access time, minutes**	11.33±8.84	10.27±7.15	0.59
**Nephroscopy time, minutes **	43.82±31.01	33.61±18.30	0.10^b^
**Overall operation time, minutes **	80.61±21.28	65.91±20.60	**0.02** ^b^
**The calyx accessed to**			0.57
**lower**	16(48.5%)	18(54.5%)	
**Middle**	7(21%)	9(27.3%)	
**upper**	9(27.3%)	6(1.2%)	
**pelvis**	1(3.3%)	0	
**Attempts for dilation **			
**one**	28(84.8%)	29(87.9%)	0.19
**two**	5(15.2)	2(6.1)	
**three**	0(0.00)	2(6.1)	
**Short dilation **	19(57.6)	12(36.4)	**0.08** ^e^
**Number of accesses **			1.00^c^
**single**	31(93.9)	30(90.9)	
**Two or more**	2(6.1)	3(9.1)	
**Exit Strategy**			.89
**Tubeless**	15(45.5)	15(45.5)	
**Nephrostomy placement**	5(15.2)	6(18.2)	
**Double-J insertion**	12(36.4)	10(30.3)	
**Both Double-J and Nephrostomy**	1(3.0)	2(6.1)	
**Early Stone Free Rate (SFR)** ^**d**^	29(87.9)	24(72.7)	0.12^e^
**Hemoglobin drop (g/dl)**	-1.76±1.49	-1.83±1.59	0.85^b^
**Renal function **			0.79^e^
**stable**	15(45.5)	14(42.4)	
**improved**	14(42.4)	13(39.4)	
**deteriorated**	4(12.1)	6(18.2)	
**Estimated GFR decrease (mL/min/1.73 m** ^**2**^ **)** ^**f**^	20.3±7.0	20.6±5.3	0.97^b^
**Duration of hospitalization**	2.39±1.47	1.97±0.91	0.30^a^
**Cost (USD)**	603±85	718±78	**0.0001**

^a^Mann-Whitney.  ^b^Independent sample T-test.  ^c^Fisher exact test.  ^d^Before additional treatment including stones less than 4 mm.  ^e^Chi-square test.  ^f^In cases with deteriorated renal function.

Data presented as frequency (column %) or mean ± SD.

**Table 4:** Complications of UPCN using Amplatz or balloon according to the Clavien-Dindo grading system.

***Complications***	***Amplatz (n=33)***	***Balloon (n=33)***	***P***
***0*** * (no complication)*	22(66.7)	22(66.7)	1
***I*** * (deviation from normal postop)*			
*Fever more than 38.5*°*C*	7(21.2)	6(18.2)	0.75
*Transient Cr rise *	1(3.0)	0	0.31
***II*** * (drugs other than grade I)*			
*Transfusion *	5(15.2)	4(12.1)	0.72
*Urine leakage* ^*b*^	0	0	NA
*Urinary tract Infection*	1(3.0)	0	0.31
***IIIa*** * (requiring local anesthesia)*			
*Hydrothorax*	0	1(3.0)	0.31
*Double-J insertion*	1(3.0)	0	0.31

a: patients with more than one complication are reported separately in each subgroup.

b: urine leakage more than 48 hours after removing the nephrostomy tube.

Data presented as frequency (column %) or mean ± SD.

NA=not applicable.

## Data Availability

The data underlying this study is available in the Shahid Bahonar Hospital Database, Kerman, Iran.
